# Safe surgical technique: iliac osteotomy via the anterior approach for revision hip arthroplasty

**DOI:** 10.1186/s13037-014-0032-7

**Published:** 2014-09-09

**Authors:** Navid M Ziran, Sherif M Sherif, Joel M Matta

**Affiliations:** 1 Hip & Pelvis Institute, Saint John’s Health Center, 2001 Santa Monica Blvd, Suite 760, Santa Monica 90404, CA, USA

**Keywords:** Hip, Arthroplasty, Revision, Anterior approach, Iliac, Osteotomy

## Abstract

Robert Judet first performed hip arthroplasty via the anterior approach (AA) in 1947 on an orthopaedic table. Our center has a near 20-year experience on more than 3500 patients operated by AA hip arthroplasty. While primary AA total hip arthroplasty techniques have been discussed in the literature, revision AA total hip arthroplasty techniques are relatively new. The current article in the Journal’s “Safe Surgical Technique” series describes the successful application of an adjunctive iliac osteotomy to improve femoral exposure in two selected patients undergoing AA revision hip arthroplasty. The potential risk/complications of an iliac osteotomy include iatrogenic fracture, malunion/nonunion, infection, and pain. These potential risks should be weighed against the potential benefits of improved surgical exposure and/or risks of other revision techniques. Future prospective longitudinal studies will be helpful to determine efficacy and risk profile compared to other revision techniques.

## 1 Background

Robert Judet performed the first hip arthroplasty on an orthopaedic table (Judet-Tasserit table) through the anterior approach in 1947 at Hospital Raymond Poincaré in Garches, France [1]. Judet termed the approach the Hueter interval and utilized the intermuscular plane between the sartorious and the tensor fascia lata. The senior author began performing total hip arthroplasty via the anterior approach (AA) in 1996 on a special orthopaedic table which was derived from the original Judet-Tasserit table. The results of the first 437 consecutive, unselected patients who had 494 primary total hip arthroplasty surgeries done through the AA were published in 2005 [2]. Other groups have also published positive outcomes on large series of patients after AA total hip arthroplasty [3],[4]. More recently, revision total hip arthroplasty via AA has become an area of investigation.

Mast et al. [5] and Kennon et al. [4] have both reported positive results using the Hueter interval for revision hip arthroplasty. This article, however, expands on the Levine modification (proximal extension of the AA) discussed in the Mast article and demonstrates a method to improve proximal femur exposure for revision surgery. In most cases, revision of the acetabular cup via AA is not as challenging. Femoral exposure, however, can be challenging due to either broach obstruction by the anterior superior iliac spine (ASIS)/ilium, large musculature, and/or a poor trajectory down the axis of the femur. For cases in which an AA primary hip arthroplasty was performed and the femoral component fails, many surgeons feel more comfortable with revision surgery via the posterior approach. If the surgeon desires to revise the femoral component via the AA, there are described techniques to facilitate femoral exposure and/or implant removal. To improve femoral exposure, the surgeon can extend the AA proximally and detach the tensor. Other techniques to aid in femoral implant removal include distal AA extension with femoral corticotomy, extended trochanteric osteotomy, and anterior femoral osteotomy (verbal communication, J. Matta, M.D.). The purpose of the iliac osteotomy is to improve exposure of the proximal femur via the AA with minimal soft-tissue morbidity. Since bony healing tends to be less morbid than soft tissue healing (i.e. tensor detachment), the authors feel this technique is an option to facilitate exposure for AA revision hip arthroplasty.

## 2 Surgical technique

**Figure 1:**
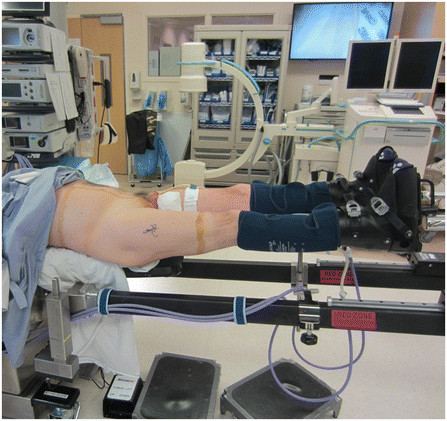
**Supine patient positioning on the hana® table with the perineal post.** The lower extremities are placed in 15 degrees of internal rotation and 5–10 degrees of hip flexion.

The patient is placed supine on the hana® table (OSI, Union City, California) as shown in Figure 1. Both lower extremities are placed in well-padded boots, which are affixed to the orthopaedic table spars. Sequential compression devices are applied to both lower extremities. A well-padded perineal post is placed, and the patient should be centered over the post. The gross traction is locked on both lower extremities. Prolonged traction (>120 minutes) should be avoided to prevent complications such as pudendal nerve palsy [6]. The lower extremities are placed in approximately 15 degrees of internal rotation and 5–10 degrees of hip flexion. The hip is then prepped and draped in sterile fashion.

**Figure 2:**
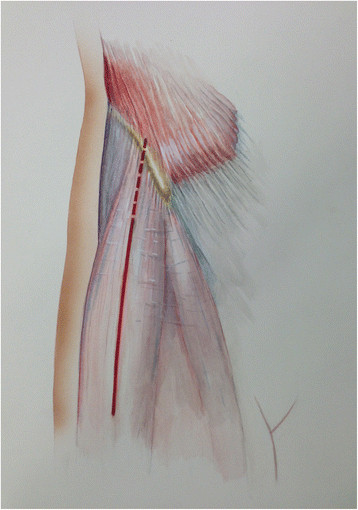
Line art drawing of the anterior approach (AA) to the hip with proximal extension lateral to the ASIS.

**Figure 3:**
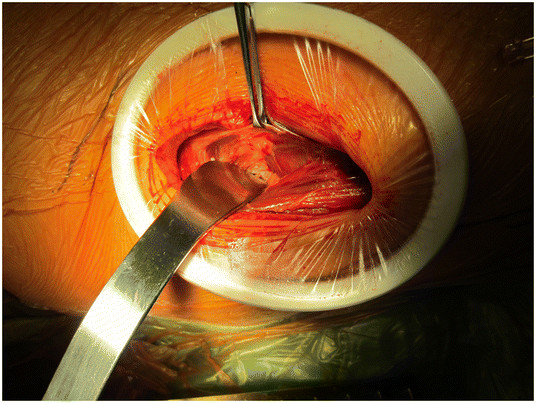
Placement of a Cobra retractor lateral to the neck capsule and medial to the tensor and gluteus minimus.

**Figure 4:**
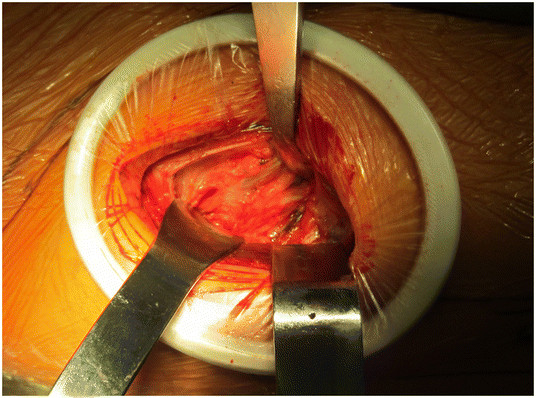
Final exposure of the anterior hip capsule with a Cobra lateral to the neck capsule and a Hohman retractor posterior to the indirect head of the rectus and medial to the neck.

An AA to the hip is performed as follows. The skin incision is based approximately 3 cm posterior and 1 cm distal to the anterior superior iliac spine (ASIS) with later proximal extension slightly lateral to the ASIS (Figure 2). The incision is centered over the tensor muscle which can be more easily palpated with the leg in internal rotation. The translucent fascia over the tensor is identified and incised longitudinally. The tensor muscle is bluntly dissected and retracted laterally. A Cobra retractor is placed lateral to the neck capsule and medial to the tensor and gluteus minimus (Figure 3). To place the medial retractor, the plane between the indirect head of the rectus femoris and the anterior neck capsule is identified and dissected sharply or with cautery. A Hohman is placed under (posterior) to the indirect head and anterior to the hip capsule. The anterior hip capsule is now exposed (Figure 4). An L-capsulotomy is performed. The leg is externally rotated, and the inferomedial joint capsule, which can sometimes impede dislocation, is excised off the inferomedial neck. The neck capsule frequently has significant granulation tissue if previously exposed from an anterior approach; this granulation tissue should be excised to facilitate dislocation.

The hip joint is then dislocated using gentle traction and external rotation. The head is removed by “tapping” it off the trunion. Another method to remove the head is *in situ* removal of the head with the joint reduced. In this method, the hip is left reduced and the head is impacted off the neck using the native cup as counter-pressure. The neck is then “pulled” out from the head. After head removal and dislocation, the hook is placed near the vastus ridge under the proximal femur with the leg in internal rotation.

**Figure 5:**
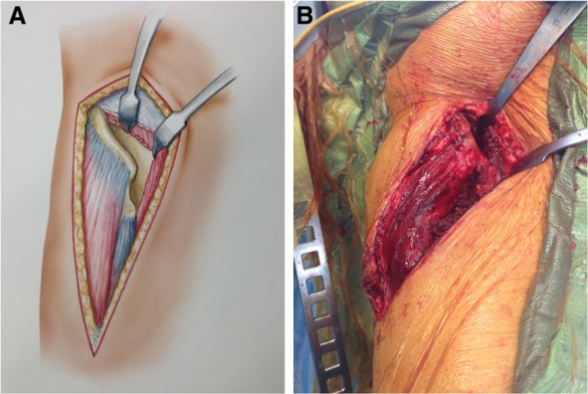
**Line art drawing (A) and photograph (B) demonstrating the deep dissection of the revision AA.** The external abdominal oblique aponeurosis has been removed off the crest down to the ASIS. The interspinous notch is bluntly dissected to the AIIS. The iliacus is bluntly dissected off the internal iliac fossa to expose the ilium.

The leg is externally rotated so the patella faces approximately 90 degrees outward. The extremity is then extended and adducted. *The surgeon should ensure that gross traction is off during this maneuver.* The hook is attached to the post and elevated to provide adequate tension. Retractors are placed around the posteromedial neck and greater trochanter, respectively. The proximal femur and the prosthesis should be exposed at this point. The old femoral head is removed via instruments at the discretion of the surgeon. At this point, access down the axis of the femur for stem removal can be challenging. The incision is extended proximally straight over the iliac crest, slightly lateral to the ASIS (Figure 2). Sharp dissection is carried down to the iliac crest. The insertion of the external abdominal oblique aponeurosis is incised off the outer crest. Care should be taken to remove the aponeurosis as it inserts around the lateral aspect of the iliac crest; the surgeon should also avoid injury to the origin of the tensor or the iliotibial band. The iliacus muscle is then bluntly dissected off the internal iliac fossa. The sartorius muscle and inguinal ligament are detached from the ASIS and blunt dissection is continued down into the interspinous notch to the anterior inferior iliac spine (AIIS) (Figure 5A-B). The lateral femoral cutaneous nerve is not exposed during the dissection. The surgeon should avoid detachment of the direct head of the rectus femoris from the AIIS.

**Figure 6:**
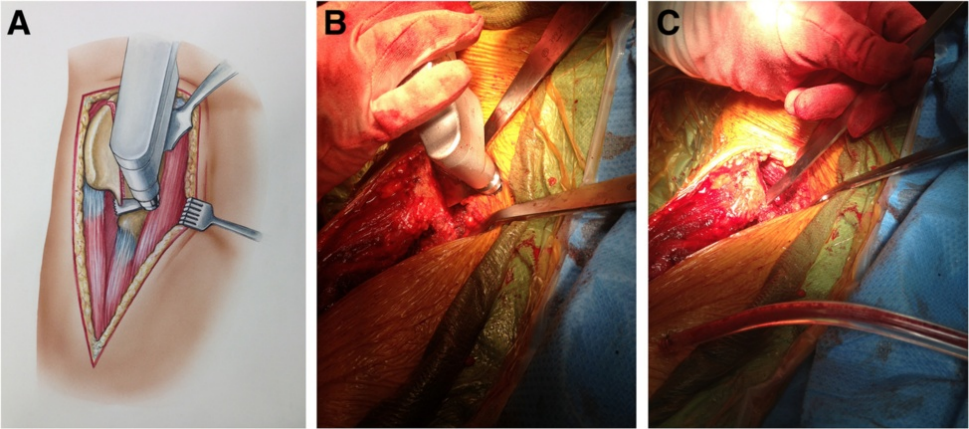
**Line art drawing (A) demonstrating the iliac osteotomy using the oscillating saw.** The osteotomy is performed first with the saw and completed with the osteotome **(B-C)**. The osteotomy should extend to the anterior border of the AIIS.

**Figure 7:**
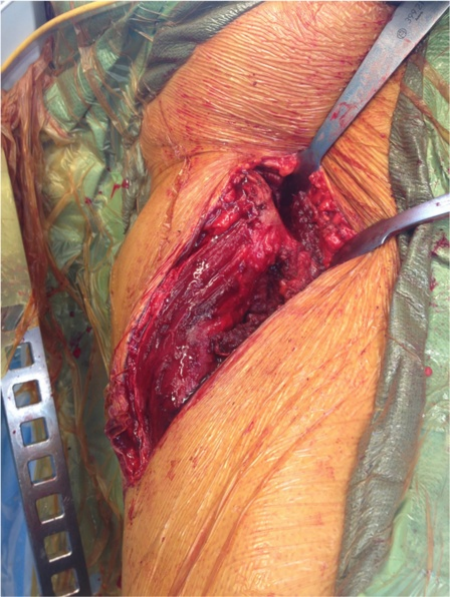
Once completed, the iliac osteotomy, with the attached tensor origin, is retracted posteriorly.

**Figure 8:**
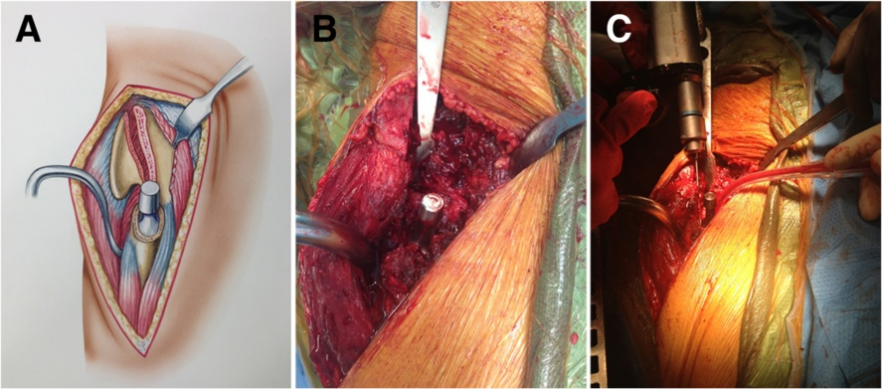
**Exposure of the proximal femur is shown with the hook placed under the vastus ridge (A).** With the osteotomy out of the way, the proximal femur is exposed and accessible **(B-C)**.

**Figure 9:**
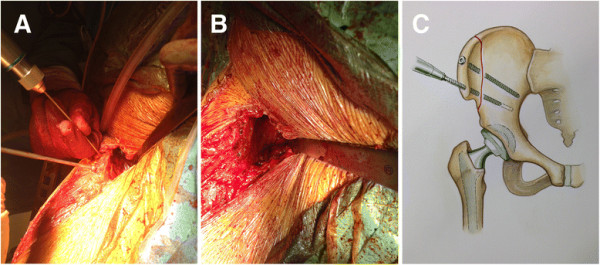
The osteotomy is reduced and fixation is achieved using two 3.5 mm cortical screws (A-C).

**Figure 10:**
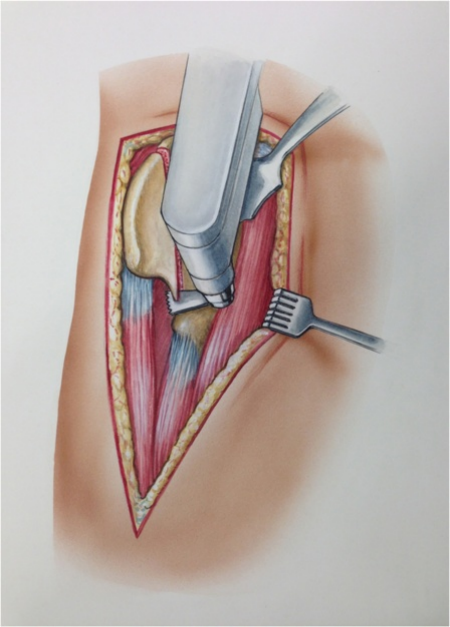
**The inguinal ligament and sartorius are repaired to the ASIS through a drill hole with #1 Vicryl suture.** This running suture is continued proximally to repair the external abdominal oblique aponeurosis to the gluteal fascia. The fascia over the tensor is also repaired using running absorbable suture (not shown).

Once the iliac fossa is exposed, the osteotomy is made. An oscillating saw is used to cut the iliac crest “inside-out” as shown in Figure 6A-B. An osteotome is used to complete the osteotomy of the outer cortex of the ilium (Figure 6C). The iliac fragment should be approximately 70 mm long and 25 mm from the crest. The osteotomy should extend from the lateral ridge of the iliac crest (approximately 5–6 cm proximal to the ASIS) to the anterior border of the AIIS. The AIIS should not be included in the osteotomy, and the direct head of the rectus femoris should be protected. The osteotomized ilium with the attached tensor muscle is then gently retracted posteriorly (Figure 7). This exposure allows for adequate access to the proximal femur as well as a facile trajectory down the femoral canal (Figure 8A-C). Once the revision is completed, the osteotomy is repaired using two 3.5 mm cortical screws (Figure 9A-C). The sartorius and inguinal ligament attachment are repaired to the ASIS using a drill hole and #1 Vicryl suture. The external abdominal oblique aponeurosis is repaired to the fascia lata using #1 Vicryl running suture (Figure 10). The fascia over the tensor is repaired using #1 Vicryl running suture. The skin is closed with 2–0 Vicryl running suture, 3–0 Monocryl, and Dermabond.

## 3 Technical tricks

1. Exposure - frequently, there is a crossing vein overlying the tensor fascia which provides an anatomic cue to the surgeon.

2. Exposure – the lateral hip capsule retractor is placed in a “pocket” distal and lateral to the anterior inferior iliac spine.

3. Exposure - attention should be spent on thorough exposure and release of the neck capsule. If a previous anterior approach was performed, this portion of the surgery can be a limiting factor due to granulation tissue. Specific attention should be paid to release of the neck capsule off the intertrochanteric line to the inferomedial neck.

4. Dislocation - the patient should have complete muscle relaxation. Dislocation can be performed by either method described in the text. Head removal can also be challenging, and the surgeon should ensure that the appropriate instrumentation is available in the OR prior to the procedure.

5. Iliac osteotomy – a complete iliac osteotomy should be ensured utilizing an osteotome from the internal iliac fossa (Figure 6C) to prevent iatrogenic fracture.

## 4 Case study #1

**Figure 11:**
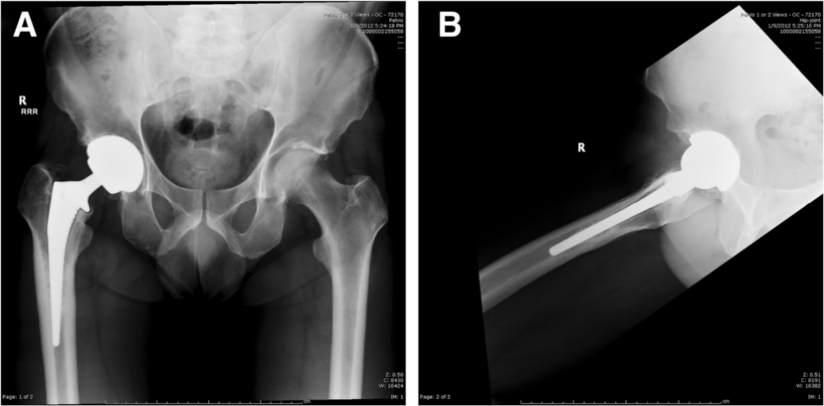
Pre-operative anteroposterior (AP) and lateral hip radiographs (A-B) demonstrating metaphyseal loosening of the femoral component.

**Figure 12:**
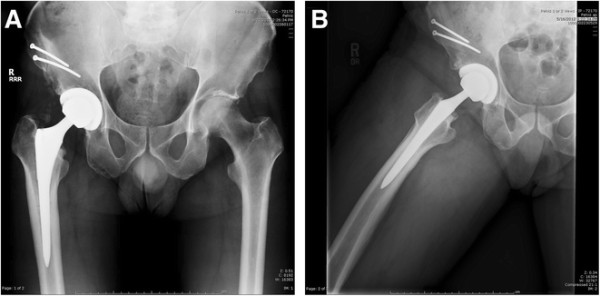
Post-operative anteroposterior (AP) and lateral hip radiographs (A-B) demonstrating the revision femoral stem/head and repair of the iliac osteotomy using two 3.5 mm cortical screws.

In the first case, the patient complained of several months of right thigh pain; the primary AA THA was approximately 4 years prior. Radiographs demonstrate proximal loosening of the femoral component in the metaphyseal region (Figure 11A-B). Revision AA hip surgery was performed through the AA and an iliac osteotomy was performed. Kirschner wires were used to facilitate removal of the femoral component distally (example shown in Figure 8C), and the femoral component was removed quite easily. The femoral prosthesis and femoral head were exchanged. Post-operative radiographs are shown approximately 3 months post-operatively (Figure 12A-B). The patient had complete relief of thigh pain and no morbidity from the iliac osteotomy. The patient was seen at one year post-operatively with no subjective pain during ambulation.

## 5 Case study #2

**Figure 13:**
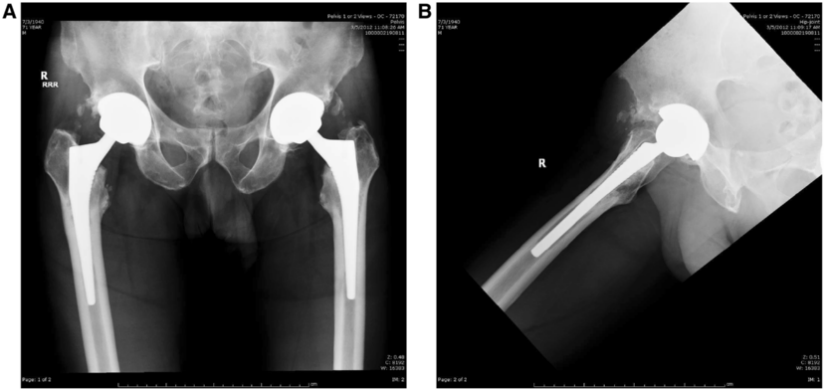
Pre-operative anteroposterior (AP) and lateral hip radiographs (A-B) demonstrating metaphyseal loosening of the femoral component.

**Figure 14:**
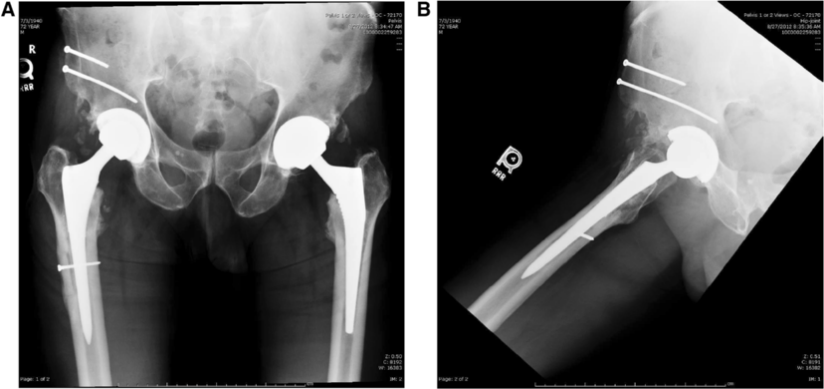
Post-operative anteroposterior (AP) and lateral hip radiographs (A-B) demonstrating the revision femoral stem/head, repair of the iliac osteotomy using two 3.5 mm cortical screws, and repair of the femoral corticotomy with a 3.5 mm cortical lag screw.

In the second case, the patient also complained of right thigh pain; the primary AA THA was performed approximately 4.5 years prior. Radiographs demonstrate proximal loosening of the femoral component in the metaphyseal region (Figure 13A-B). Revision hip surgery was performed through the AA and an iliac osteotomy was performed. Kirschner wires were utilized to facilitate distal femoral component removal, as in case 1, but were not successful. In this case, the approach was extended distally and a subvastus approach was performed to the lateral femur. A femoral corticotomy was performed to allow exposure of the distal portion of the femoral component. Osteotomes and Kirschner wires were utilized via the bone window to facilitate removal of the femoral component. Six-month post-operative radiographs are shown (Figure 14A-B) and demonstrate consolidation of the iliac osteotomy. The patient had complete relief of thigh pain at 6 and 14 months post-op.

## 6 Discussion

Revision anterior approach arthroplasty is a field in evolution as techniques to facilitate exposure and extract implants are introduced. Such techniques include: proximal extension of the anterior approach with detachment of the tensor origin on the iliac crest, distal extension of the incision with a femoral corticotomy, or an extended trochanteric osteotomy. The index procedure discusses proximal extension of the incision with exposure of the internal ilium. The purpose of the iliac osteotomy is to facilitate exposure and instrumentation of the proximal femur that would otherwise be impeded by the ilium and/or a large tensor muscle. In cases where the ilium is a mechanical impediment to the broach handle, dual-offset broaches handles have been utilized although they are not co-axial with the femoral canal.

In the described technique, the osteotomized ilium is retracted posteriorly along with the tensor attachment; the proximal femur is thereby exposed with no mechanical impedance either to the proximal femur or to a straight, co-axial trajectory down the femoral canal. Compared to detachment of the tensor as a strategy for femoral exposure, the osteotomy provides relief from both soft tissue and bone impediments while not disrupting the soft tissues of the hip deltoid. Soft-tissue morbidity associated with tensor origin detachment may compromise hip function (flexion, abduction) versus the high union rate of an iliac osteotomy.

If, during revision of the femoral component, it is necessary to expose the distal femur, the incision can be extended distally. The deep dissection can be performed posterior (subvastus) to the vastus lateralis with cauterization or ligation of the venous perforators near the bone. The vastus lateralis is elevated anteriorly and the distal femur is exposed. Iatrogenic fracture fixation can be performed with this extended approach. If cement removal is necessary, the surgeon can perform either a corticotomy or an extended trochanteric osteotomy through this approach.

There are limitations of this technique. First, the surgeon should be comfortable with the anatomy around the pelvis and acetabulum. While not part of the approach, the lateral femoral cutaneous nerve runs lateral in the sartorious, and if the surgeon is not careful detaching the sartorious/inguinal ligament off the ASIS, it can be injured. Next, the iliac osteotomy should include the origin of the tensor – anterior to the gluteus medius tubercle extending into the interspinous notch as shown in Figure 5B. Iatrogenic fracture can occur if the osteotomy is not completed. The external abdominal oblique aponeurosis and sartorious/inguinal ligament should be adequately repaired to the tensor fascia lata and ASIS, respectively. Because we have only described two cases with limited follow-up, the potential for other complications is unknown.

## 7 Conclusion

This article describes a technique, which may aid the surgeon in performing revision of the femoral component through the AA. The authors recognize that femoral component revision surgeries are not always best performed via the AA; this article describes a potentially helpful technique via the AA to facilitate femoral exposure and minimize soft-tissue compromise. Hip capsule release, muscle relaxation, and understanding of pelvic anatomy are key aspects of this procedure. Further prospective studies on this technique should be performed to determine its morbidity and efficacy compared to other revision techniques.

## 8 Consent

Written informed consent was obtained from the patients for publication of this article.

## Competing interest

The authors declare no other competing interests related to this manuscript. Each author certifies that he has no commercial associations (e.g. consultancies, stock ownership, equity interest, patent/licensing arrangements, etc.) that might pose a conflict of interest in connection with the submitted article.

## Authors’ contributions

NMZ and SS provided the drafts of the manuscript. NMZ provided the intraoperative pictures. JMM and NMZ commissioned the line-art. JMM innovated the technique, performed the surgeries, edited the manuscript, and provided oversight. All authors read and approved the final version of this article.
